# Notes, Queries and Formulæ

**Published:** 1878-10-20

**Authors:** 


### *Practical Notes and Formulae.*

### FELONS.

A writer in *Physio Medical Journal* says: for felons in the palm of the hand. I prepare a compound as follows :

℞ Lobelia Sem......................dr jj.
Cypripedium.
Ginger ..........................aa dr j.
Capsicum.........................dr ss.
Ulmus F..........................dr iv.
Powdered and mixed.

First bathe the hand in good strong lye, as hot as can be borne, for five or ten minutes; then apply a hot poultice made of the above compound and mixed to a proper consistence with the lye, wetting the poultice every hour with a few drops of the strong tincture of lobelia seed. Change the poultice morning, noon and night, and each time before applying the new poultice bathe well the affected parts as at first in hot lye. One heap ing teaspoonful of the above compound is sufficient for each poultice and the use of the tincture of lobelia for twenty-four or thirty-six hours will also be sufficient. After the first few hours there is comparatively but little pain, the difficulty rapidly terminates leaving no stiffened or crippled hands or fingers. This is my experience gathered from the treatment of many cases. After the sack loosens and is extracted, then heal with some good healer.

*Smartweed.*—The smartweed, "Polygonum Punctatum," P. hydropiperoide, is a common weed, well known as a domestic remedy; but its true place in therapeutics is but little known or valued. In dysentery, watery and mucous diarrœha, few agents have proven of more value. The dry plant yields about eighteen per cent. tannin. What its other proximal principles are I do not know. I prepare a saturated tincture. As an anti-pyretic in typhomalarial and our bilious remittent fevers, it has few eqauls. With it the tempature may, in a few hours, be brought down from 102°, or even 104°, to 98°, and at the same time it is valuable as an anti-periodic. It is an active stimulant, and combined with some of the salts of the cinchona, one-half the ordinary dose of the latter will have a better effect than a full dose uncombined.

When taking polygonum, the patient feels it to be a stimulant, and will say, "that medicine warms me all over, I feel it to the tips of my fingers." In dysenentery, when there is tormina and tenesmus, I give from m. xx to dr. i in mucilaginous drinks every hour or two, as occasion demands; and when the discharges are so fiequent as to prostate and cause loss of sleep, an enema of polygon., gr i in an ounce of cold water, after each dejection, will calm and still the bowels in a short time. In hæmorrhage of the bowels, no drug within my knowledge given by the mouth and an enema will so soon check it. I think it has no, or but little, influence in reducing the rates of the pulse. I use it in all forms of bowel complaints, particularly in the diarrhœa of typhoid fevers. It acts as a diaphoretic and mild diuretic.

For the watery diarrhœa for children I use—

℞ Tinct. Polygon....................dr i.
Tr. rhœi comp
Tinct. zinziber...................aa dr. iv.
Tr. camphor.......................dr. ij.
Syr. anise........................dr. vi.
Sodæ bicarb.......................dr. i.

*Sig*:—From half to a teaspoonful, according to good judgment. This formula having no opium in it is a safe domestic remedy.

### Glycerite of the Hypophosite of Zinc.

*Dr. Polk in The Druggists Circular says*: About eighteen months ago I wished to use hypophosphite of zinc in a very obstinate case of chorea, and failing to obtain it from any of the manufacturing chemists I improvised the following formula:

R Sol. hypophosphorous acid (50 per cent. terhydrated)..............oz. iv.

Zinci corbonatis...... ................ q. s.

Aquæ...... ...................... .. q. s.

Glycerine (C. P.)......q. s. ad fl. oz xvj.

Rub the zinc to a paste with water, add to the hypophosphorous acid nearly to saturation, filter and add the glycerine to the required amount.

Of this glycerite I administered ten drops thrice daily in water to a girl thirteen years of age, curing the case of chorea which had defied all other treatment. The rapid improvement in the general health (which yet remains excellent) in this case induced me to use it in one of hysteria with satisfactory results. I am confident it will be found superior to all other zinc preperations in nervous dieases.

### ANTIDOTE TO CARBOLIC ACID.

The Pharmaceutishe Zeitung fur Russland says that on the recommendation of Professor Baumon, Dr. Sanftleben has used sulphuric acid in several cases of poisoning by carbolic acid with the best success, the phenol combining with the acid to form phenyl-sulphuric acid, which is not poisonous. He administered it in a mixture composed of dilute sulphuric acid 10.0, mucilage of gum 200.0, and a simple syrup 30.0 grams, in doses of a tablespoonful every hour.

### IODINE IN INTERMITTENTS.

Dr. Schell, in Cin. *Lancet*, claims good success with this remedy, used as ollows:

R. Tinct. Iodii..................gtt. 60.
Iodide Potassa................grs. x.
Syr. Simplex,.................ozj. M.

Sig. One teaspoonful half hour after each meal.

After a careful study of the pathology of intermittent fever and the action of iodine in the system, it seems to me that it is a remedy that a great amount of good may be expected from in malarial diseases.

*Common Salt as a Remedy for Intermittent Fever.*—Dr. Brokes recommends for periodic fevers a very simple method of treatment, the efficacy of which he has often demonstrated in his travels in America and Hungary. He takes a good handful of table salt and roasts it in a clean, and, if possible, new, pan over a gentle fire, until it assumes a brown color, like that of roasted coffee. When the patient is a vigorous adult, a good tablespoonful of this salt should be desolved in a glass of warm water, and the whole swallowed at a dose. When the fever recurs at intervals of two, three or four days, the remedy should be taken fasting on the morning after the day of the paroxysm. When the thirst excited by the salt becomes insupportable, a little water may be allowed to the patient, but it should be sucked through a straw. For forty-eight hours after the remedy has been taken no food should be allowed, except some beef or chicken soup; it is particularly necessary to observe a strict diet and to avoid chilling of the body during this period. Dr. Brokes asserts that he has employed this method of treatment for eighteen years, and invariably with success.—*Gazette des Hopitauex.*

Perfumed Carbolic Acid.

Carbolic acid.................. 1 part.

Oil lemon...................... 3 parts

Alcohol at 36°.................100 parts

The oil of lemon completely masks the disagreeble order of the acid, without at all interfering with its properties. The above was long a secret preperation, known as Lebon's Perfumed Carbolic Acid

*Antidote to Arsenic.*—It has been noted by Rouyer that freshly precipitated sesquihydrate of iron, although an antidote for arsenious acid (arsenic of the shops), fails entirely to counteract the action of arseniate of soda or arsenite of potassa (Fowler's solution), but that a mixture of a solution of the sesquichloride of iron and the oxide of magnesium will counteract the effect of these salts, as well as the arsenious acid itself, and hence this mixture is always to be preferred to the hydrate in cases of poisoning by arsenic. The official solution of the sesquichloride of iron should first be administered, and afterwards the magnesia. In one hour after the administration of the antidote, a cathartic should be given. In all cases acid drinks (such as lemonade) are to be avoided, since the compounds they form are soluble.—*New Remedies.*

*Chlorodyne* (*G. H. F. Toledo, Ohio.*)—The formula which is supposed to approach nearest to the original preparation of Dr. J. Collis Brown is that given by Squire in his companion to the Brit. Pharm. (11th ed., p. 96) This is as follows:

| | |
|---|---|
| Chloroform | fl. oz. 4. |
| Ether | fl. oz 1. |
| Alcohol | fl. oz. 4. |
| Molasses | fl. oz. 4. |
| Extract of licorice | fl. oz. 2½. |
| Morphia hydrochlorate | gr. 8. |
| Oil peppermint | m.16 |
| Hydrocyanic acid (2 per cent) | fl oz. 2 |
| Syrup | fl. oz . 17½ |

Dissolve the morphia salt and the oil of pepermint in the alcohol; mix the chloform and either with this solution; dissolve the extract of licorice in the syrup and add the molasses; shake these two solutions together and add the hydrocyanic acid.

We would reccommeud to use only purified extract of licorice, of soft consistency and entirely soluble, to make this preparation, as the amount of deposit will thereby be considerably reduced. There have been a great many formulæ of this preparation published. We have about eighteen different ones on file; but as we ourselves prefer that given above, we do not wish to induce others to make use of different ones.

*Postage-Stamp Mucilage* (*Hawkins Tex.*)

The composition of this is *said* to be:

| | |
|---|---|
| Dextrin | 2 parts. |
| Acetic acid | 1 part. |
| Water | 5 parts. |

Dissolve in a water-bath and add

| | |
|---|---|
| Alchohol | 1 part;— |

but we are informed that another mixture is used at present. We will endeavor to learn its composition.—*New Remedies.*

### TO REMOVE FOREIGN BODIES FROM THE NOSE.

Dr. John Winslow, of Ithaca, New York, sends us the following directions to be pursued in this emergency: "Close the empty nostril by pressure with your finger, and blow suddenly and forcibly into the child's mouth. The bean or button commonly flies out at the first effort; if not, a repetition is pretty sure to expel it."—*New Remedies.*

### PULVIS PAREGORICUS.

(O. H. N. Y.)—This powder has once been used as a solid substitute for tincture opii camphorata, and was originally proposed by Vogler. Its composition was: Starch, 80 grs.; mastic, 40 grs.; powdered opium 2 grs. The dose ranged from 5 to 30 grains.

### TONIC IN ANÆMIA.

| | | |
|---|---|---|
| R | Tinct. ferri chloridi | f. dr. iij. |
| | Potassii chloratis | dr. i· |
| | Solut. strychniæ sulph. (gr. i. ad f. oz. i.) | f. dr. ij. |
| | Syrupi simplicis | f. dr. iv. |
| | Aquæ puræ q. s. ad | f. dr. iv. |

M. Sig. Two teaspoonfuls, three times, daily, as a tonic in anæmia.—*Dr. D. S. Brainard.*

